# Catarrh of the Uterus

**Published:** 1870-05

**Authors:** G. W. Hamilton

**Affiliations:** Orangeville, Ohio


ART. II.—*Catarrh of the Uterus.* By G. W. HAMILTON, M. D., *Orangeville, Ohio.*

Catarrh of the uterus, uterine leucorrhea, inflammation of the lining membrane of the uterus, followed by congestion, and terminating in hemorrhage (menorrhagia,) or suppuration (leucorrhœa,) may terminate just as inflammation in any other organ. In young females, of a delicate constitution, it is common to find a secretion of mucus at one or two monthly periods preceding the development of the catamenia. This discharge will frequently continue after the development of the catamenia, between the menstrual periods. It produces great debility by its continual drain upon the system, and impoverishes the blood in the same manner that a chronic ulcer, in any part of the body, would. If no treatment is adopted, it may continue for years. The principal, predisposing causes, are a scrofulous habit, want of, or, too much exercise, improper food, late hours, insufficient clothing, wet feet, and anxiety of mind. The literature of the present day, producing by a morbid state of the mind, is a very common cause. The principal exciting cause is irritation. This may be from cold, abortion, child-bearing, irritating injections to cure some fancied disease, or onanism. Onanism is the principal cause in young females; as we verily believe this is practised to as great an extent among females, as males. It is a delicate subject, but it is time that something more was done to prevent this habit, which is sending more of the young of our country to the grave and insane asylums, than all other causes combined. Let us leave this class of patients to empirics no longer; let not the physician be too delicate to expose the danger of this body-destroying and soul-ruining habit. Young females should be permitted to exercise freely in the open air; and run, and romp, like boys.

*Symptoms.*—A certain degree of languor, some weakness of the back, headache, pale complexion, a sensation of weight in the pelvis, and, occasionally, of bearing down. We often find associated with it, itching of the genitals, extending up into the vagina. The patient soon becomes debilitated, the pulse weak, the skin sallow, and the eyes sunken, with a dark circle around them. The tongue is clean and flabby, the papilla not very prominent. By an examination per vaginum, we find the os a very little dilated. The discharge varies in quantity; it being sometimes very profuse, and sometimes scanty.

*Diagnosis.*—This disease is sometimes mistaken for gonorrhœa. In gonorrhœa there is a burning pain all through the genital canal; the discharge is of a dark color; there is scalding on passing urine, and some discharge from the urethra. In vaginal leucorrhœa the symptoms are not very severe; in fact, the patient often recovers without the aid of medicines.

*Treatment.*—This is a local disease at the beginning; but when it becomes chronic, it becomes constitutional, and requires constitutional treatment. Ergot, followed by some of the preparations of iron internally, and vaginal injections of a solution of the persulphate of iron; (twenty-four grains of the powder to one-half pint of water,) is a favorite prescription. I would not advise the use of the nitrate silver, or any other caustic, unless there is ulceration of the os; cauterizing the lining membrane of the uterus, cannot but be fraught with danger. The uterus is a tender organ, but it does endure much more than we would suppose that it could. Sponge the back and lower part of the abdomen with tepid water twice a day. In cold weather require the patient to wear flannel next the skin; keep the bowels regular with some mild aperient, followed by an enema, if necessary. Fld. ext. taraxaci, will answer very well. Opium or conium may be given if there be much irritation. Fresh air, and moderate exercise, as riding in a carriage when the weather will permit. It will be asked how does the ergot act? It produces contraction of the uterus and capillaries. In catarrh of the uterus we have congestion, and then inflammation, followed by suppuration and dilatation of the capillaries. By giving the ergot first, we get a healthy action of the circulation. As ergot does not retard the circulation to a very great extent unless by continued use, it may be given a long time in proper medicinal doses, and without any injurious effects. The common opinion that ergot, if long continued, will produce dry gangrene, is an erroneous one; ergot is one of the best remedies for congestion or hemorrhage that we possess. In a chronic case of this disease I would recommend the fld. ext. ergot three times a day for three or four weeks, and longer if necessary; after which, would give some preparation of iron.

The common practice of cauterizing the lining membrane of the uterus is very injurious, but vaginal injections of a solution of the persulphate of iron are beneficial. In ulceration of the os a strong solution of the persulphate, applied with a camel hair pencil, will be found as beneficial, in most cases, as the caustic; but there are some cases which require more active remedies. Much attention should be given to moderate exercise, and the avoidance of exciting causes. Let the patient commence with exercise in an easy carriage, followed by walking and horseback riding; give her cheerful company, and moral and entertaining reading. Plain food, corn or Graham bread, weak tea and coffee, boiled rice, &c. We pay too little attention to the diet in these cases. If the patient tire of one preparation of iron, give her another, alternate with the ergot.

This is a brief outline of my mode of treatment, and of the views I entertain of the nature of this malady.

ORANGEVILLE, Ohio, March 31, 1870.

